# Diosmin ameliorates renal fibrosis through inhibition of inflammation by regulating SIRT3-mediated NF-κB p65 nuclear translocation

**DOI:** 10.1186/s12906-023-04330-z

**Published:** 2024-01-09

**Authors:** Wen-Man Zhao, Xun-Liang Li, Yuyu Zhu, Rui Shi, Zhi-Juan Wang, Jian-Ping Xiao, De-Guang Wang

**Affiliations:** 1 Department of Nephrology, the Second Affiliated Hospital of Anhui Medical University, 678 Furong Road, Hefei, 230601 Anhui China; 2 Institute of Kidney Disease, Inflammation & Immunity Mediated Diseases, the Second Affiliated Hospital of Anhui Medical University, Hefei, China

**Keywords:** Diosmin, Fibrosis, Inflammation, NF-κB p65, UUO, SIRT3

## Abstract

**Background:**

Renal fibrosis is considered an irreversible pathological process and the ultimate common pathway for the development of all types of chronic kidney diseases and renal failure. Diosmin is a natural flavonoid glycoside that has antioxidant, anti-inflammatory, and antifibrotic activities. However, whether Diosmin protects kidneys by inhibiting renal fibrosis is unknown. We aimed to investigate the role of Diosmin in renal interstitial fibrosis and to explore the underlying mechanisms.

**Methods:**

The UUO mouse model was established and gavaged with Diosmin (50 mg/kg·d and 100 mg/kg·d) for 14 days. HE staining, Masson staining, immunohistochemistry, western blotting and PCR were used to assess renal tissue injury and fibrosis. Elisa kits were used to detect the expression levels of IL-1β, IL-6, and TNF-α and the activity of SIRT3 in renal tissues. In addition, enrichment maps of RNA sequencing analyzed changes in signaling pathways. In vitro, human renal tubular epithelial cells (HK-2) were stimulated with TGF-β1 and then treated with diosmin (75 μM). The protein and mRNA expression levels of SIRT3 were detected in the cells. In addition, 3-TYP (selective inhibitor of SIRT3) and SIRT3 small interfering RNA (siRNA) were used to reduce SIRT3 levels in HK-2.

**Results:**

Diosmin attenuated UUO-induced renal fibrosis and TGF-β1-induced HK-2 fibrosis. In addition, Diosmin reduced IL-1β, IL-6, and TNF-α levels in kidney tissues and supernatants of HK-2 medium. Interestingly, Diosmin administration increased the enzymatic activity of SIRT3 in UUO kidneys. In addition, Diosmin significantly increased mRNA and protein expression of SIRT3 in vitro and in vivo. Inhibition of SIRT3 expression using 3-TYP or SIRT3 siRNA abolished the anti-inflammatory effects of diosmin in HK-2 cells. Enrichment map analysis by RNA sequencing indicates that the nuclear factor-kappa B (NF-κB) signaling pathway was inhibited in the Diosmin intervention group. Furthermore, we found that TGF-β1 increased the nuclear expression of nuclear NF-κB p65 but had little significant effect on the total intracellular expression of NF-κB p65. Additionally, Diosmin reduced TGF-β1-caused NF-κB p65 nuclear translocation. Knockdown of SIRT3 expression by SIRT3 siRNA increased the nuclear expression of NF-κB p65 and abolished the inhibition effect of Diosmin in NF-κB p65 expression.

**Conclusions:**

Diosmin reduces renal inflammation and fibrosis, which is contributed by inhibiting nuclear translocation of NF-κB P65 through activating SIRT3.

**Supplementary Information:**

The online version contains supplementary material available at 10.1186/s12906-023-04330-z.

## Background

Worldwide mortality due to chronic kidney disease (CKD) has increased markedly over the past 25 years and continues to increase [1]. CKD has become a serious public-health problem that has caused great suffering and economic burden to people worldwide [2]. The multifaceted etiology of CKD encompasses various underlying factors, including diabetes, hypertension, inflammation, oxidative stress, and metabolic disorders [3]. However, efficacious drugs that can slow-down CKD progression are lacking. Studies have shown that the pathologic development of CKD involves progressive glomerulosclerosis, tubular constriction/dilation, death of tubular cells, and interstitial fibrosis. Among them, interstitial fibrosis [4] is a common pathway for almost all types of CKD to progress to end-stage renal disease. Renal fibrosis is now recognized as a major determinant of renal pathology [5]. Inflammation is often considered an important initial event in renal fibrosis [6–8]. Various physiologic injuries or inflammatory cytokines can trigger renal fibrosis in the initial stages and eventually cause end-stage renal disease. The sustained inflammatory response induces the release of cytokines and initiates various signaling pathways which, in turn, have a crucial role in enhanced myofibroblast activity and excessive accumulation of the extracellular matrix, and have an important pathologic basis for it [9]. Experimental studies [10, 11] have shown that use of specific anti-inflammatory measures in CKD has excellent antifibrotic and nephroprotective effects.

Silent information regulator 3 (SIRT3) belongs to a family of evolutionarily conserved deacetylases that mediate nuclear gene expression, metabolic control, the cell cycle, and cell proliferation [12, 13]. There is increasing evidence that SIRT3 plays an important part in the development of kidney disease. Increasing the expression and activity of SIRT3 can delay the development of kidney disease. Conversely, deficiency of SIRT3 leads to high levels of oxidative stress, increased chronic inflammation, mitochondrial dysfunction, and defective telomere instability [14, 15]. These cellular processes are involved in the progression of diseases such as diabetic nephropathy, renal fibrosis, and acute kidney injury, and strongly aggravate the pathologic damage to the kidney.

Diosmin is a glycosylated polyphenolic flavonoid found in *Citrus aurantium (Citrus sinensis)* and olive leaves *(Olea europaea)* [16–18]. Several clinical studies have demonstrated the anti-inflammatory potential of Diosmin [19, 20]. Diosmin can inhibite levels of TNF-α, IL-1β, and IL-6 while promoting IL-12 production in patients with chronic venous disease [19]. In preclinical studies, Diosmin has also shown a range of pharmacological activities including antioxidant, anti-inflammatory and anti-apoptotic [21]. Diosmin has been shown to attenuate paraquat-induced lung inflammation and fibrosis in mice by increasing glutathione levels and catalase activity and decreasing hydroxyproline levels and malondialdehyde levels [22]. Gerges et al. [23] demonstrated that Diosmin ameliorated inflammation, insulin resistance, and fibrosis in a rat model of non-alcoholic steatohepatitis. Furthermore, a preclinical study [24] showed that Diosmin regulates oxidative stress-mediated renal inflammation and apoptosis via the NF-kB pathway which, in turn, attenuates adriamycin-induced renal injury in rats. However, whether Diosmin provides protection against unilateral ureteral obstruction (UUO)-induced CKD by inhibiting renal inflammation and interstitial fibrosis is not known. We designed in vivo and in vitro experiments to determine the beneficial effects of Diosmin on ameliorating renal fibrosis and the underlying mechanisms involved.

## Materials and methods

### Reagents

Diosmin (cat no.: HY-N0178) and 3-TYP (HY-108331) were purchased from MedChemExpress (Monmouth Junction, NJ, USA). Human proximal tubular epithelial (HK-2) cells were obtained from American Type Culture Collection (Manaszsas, VA, USA). Fetal bovine serum was sourced from MilliporeSigma (Burlington, MA, USA). Transforming growth factor-β1 (TGF-β1), primary antibodies α-smooth muscle actin (α-SMA), collagen I, NF-κB p65, glyceraldehyde 3-phosphate dehydrogenase (GAPDH)) and secondary rabbit-binding antibodies were purchased from Proteintech (Chicago, IL, USA). Lipofectamine™ 2000 Transfection Reagent (11668019) was from Thermo Fisher Scientific (Waltham, MA, USA). A reverse transcription kit and SYBR™ Green PCR master product was purchased from Vazyme (Nanjing, China).

### Animals and experimental procedures

The protocol for animal experimentation was approved (LLSC20210354) by the Animal Care and Use Committee of Anhui Medical University (Hefei, China). The study was conducted in accordance with the Experimental Animal Administration regulations issued by the State Committee of Science and Technology of the People′ s Republic of China. All the procedures for the care of the mice were in accordance with the institutional guidelines for animal use in research. Renal fibrosis was induced by UUO surgery. UUO model is a classic and rapid model to simulate renal fibrosis [25]. In the UUO model, ligation of the ureter resulted in obstruction of the renal outflow tract on the ligation side, hydronephrosis with tubule dilation, and subsequent immune cell infiltration and fibrosis. Mechanical stretching of tubular epithelial cells leads to cell damage and death, which recruits large numbers of immune cells and causes an inflammatory response. The result is activation and proliferation of myofibroblasts. C57BL/6 mice (8 weeks; 20 ~ 25 g) were housed in SPF (Specific Pathogen Free) grade animal houses on an ad libitum diet at a temperature of 22–24 degrees Celsius and a humidity of approximately 60%. Mice were divided into four groups of five [22, 26]: sham operation (Sham), UUO, UUO + Dios-1 (Diosmin 50 mg/kg), and UUO + Dios-2 (Diosmin 100 mg/kg).

Diosmin was dissolved in dimethyl sulfoxide (0.5% *v/v*) and administered by gavage for 14 days after UUO surgery [27, 28]. Changes in bodyweight, food intake, and water intake were recorded every week. Mice were sacrificed by intraperitoneal injection of sodium pentobarbital (50 mg/kg). Serum was collected before mice were killed. Levels of alanine transaminase (ALT), aspartate transaminase (AST), alkaline phosphatase (ALP), blood urea nitrogen (BUN), and serum creatinine (Scr) were measured using an automated biochemical analyzer (Roche, Basel, Switzerland). Kidney tissue was fixed with 4% paraformaldehyde, embedded in paraffin, and sectioned to a thickness of 5 mm. Paraffin sections were stained with hematoxylin and eosin (H&E) or Masson's trichrome for morphology studies. Masson trichrome-stained fibrotic areas and positive areas were analyzed using ImageJ 1.47 (US National Institutes of Health, Bethesda, MD, USA).

### RNA sequencing analysis

The mRNA of the kidneys was extracted and sent to Gene Denovo Technology Co., Ltd. for mRNA sequencing. Differential gene expression analysis was completed using the R package limma (version 3.44.3). The fold change (FC) of each gene was log2 transformed and further analyzed using the R package clusterProfiler (version 3.16.1). Then, gene set enrichment analysis of canonical pathways was conducted to find differentially regulated pathways.

### Cell-viability assay

To assess cell viability, HK-2 cells were cultured at 1 × 10^4^ cells per well in 96-well plates. After treatment with Diosmin for 3 h, they were incubated with 0.5 mg/mL of 3-(4,5-Dimethylthiazol-2-yl)-2,5-diphenyltetrazolium bromide solution (100 μL/well) for 4 h. After washing with 1 × PBS, dimethyl sulfoxide (150 μL/well) was added to dissolve purple crystals. The absorbance of the sample was measured at 570 nm with a microplate reader (BioTek Instruments, Winooski, USA).

### Small interfering RNA (siRNA) transfection

Human SIRT3-specific siRNA was synthesized by QingkeBio (Beijing, China). The target nucleotide sequence of the siRNA was CATCGATGGGCTTGAGAGA. HK-2 cells were added to a six-well plate at 2 × 10^4^ cells/well. After cells had become attached, the medium was changed 1 h before transfection. Then, siRNA (50 nM) was transfected for 24 h using Lipofectamine 2000 (Thermo Fisher Scientific) according to manufacturer instructions.

### Elisa

Elisa kits for interleukin (IL)-6 (RX203049M), IL-1β (RX203063M), and tumor necrosis factor (TNF)-α (RX202412M) were obtained from Quanzhou Ruixin Yijian Biotechnology (Quanzhou, China). Levels of IL-1β, IL-6, and TNF-α in kidney and the supernatant of HK-2 cell-culture medium were detected using Elisa kits according to standard protocols, respectively.

### Assay to measure the activity of SIRT3

The activity of SIRT3 in mouse kidneys was measured by a SIRT3 enzyme activity detection kit (KL-SIRT3-Hu02, Shanghai, China). The activity of SIRT3 in specimens was measured by the double-antibody sandwich method. Results were obtained from three replicate experiments.

### Western blotting

Western blotting was used to measure the protein expression in HK-2 cells or mouse kidney tissue after treatment (Part of the the blots were cut prior to hybridisation with antibodies during blotting). Antibody binding was detected using a western blotting detection kit (Thermo Fisher Scientific). Captured western blotting bands using an ultrasensitive multifunction imager (AI600RGB; General Electric, Boston, MA, USA). Bands were quantified with ImageJ and normalized to GAPDH expression.

### Quantitative PCR

Total RNA was extracted from HK-2 cells or mice kidney tissue using TRIzol® Reagent. Complementary-DNA was synthesized by Hiscript Q RT SuperMix and used for a qPCR (+ gDNA wiper) reverse transcriptase kit (Vazyme). Real-time PCR was undertaken on primers, the sequences of which are shown in Table 1. Messenger (m)RNA expression of the corresponding samples was normalized with GAPDH mRNA.

**Table 1:** Primer sequences used for RT-qPCR

**Gene**	**Forward (5’ to 3’)**	**Reverse (5’ to 3’)**
Mus Collagen I	GACGCCATCAAGGTCTACTG	ACGGGAATCCATCGGTCA
Mus α-SMA	ACTGGGACGACATGGAAAAG	GTTCAGTGGTGCCTCTGTCA
Mus SIRT3	CACGTTTACAAACATGAACC	CATGCTAGATTGCCCTAGT
Mus IL-6	GAGGATACCACTCCCAACAGACC	AAGTGCATCATCGTTGTTCATACA
Mus IL-1β	GACCTTCCAGGATGAGGACA	AGCTCATATGGGTCCGACAG
Mus TNF-α	CGTCGTAGCAAACCACCAAG	TTGAAGAGAACCTGGGAGTAGACA
Mus GAPDH	ACCCAGAAGACTGTGGATGG	ACACATTGGGGGTAGGAACA
Hum Collagen I	TGACCTCAAGATGTGCCACT	ACCAGACATGCCTCTTGTCC
Hum α-SMA	CCCGGGACTAAGACGGGAAT	CCATCACCCCCTGATGTCTG
Hum SIRT3	GAGGTTCTTGCTGCATGTGGTTG	AGTTTCCCGCTGCACAAGGTC
Hum IL-6	CTGCAGCCACTGGTTCTGT	CCAGAGCTGTGCAGATGAGT
Hum IL-1β	CGATGCACCTGTACGATCAC	TCTTTCAACACGCAGGACAG
Hum TNF-α	TGCACTTTGGAGTGATCGGC	ACTCGGGGTTCGAGAAGATG
Hum GAPDH	TGATGACATCAAGAAGGTGGTGAAG	TCCTTGGAGGCCATGTGGGCCAT

*Mus* mouse, *Hum* human, *SIRT3* sirtuin 3

### Statistical analyses

Values are the mean ± SEM. Quantitative data were tested for normality. Two-tailed unpaired *t*-test and one-way ANOVA were used to compare differences between two groups and multiple groups, respectively. Prism 9.0 (GraphPad, San Diego, CA, USA) was used for statistical analyses. *p* < 0.05 was designated significant.

## Results

### Diosmin attenuates UUO-induced renal injury and inflammation

**Fig. 1:**
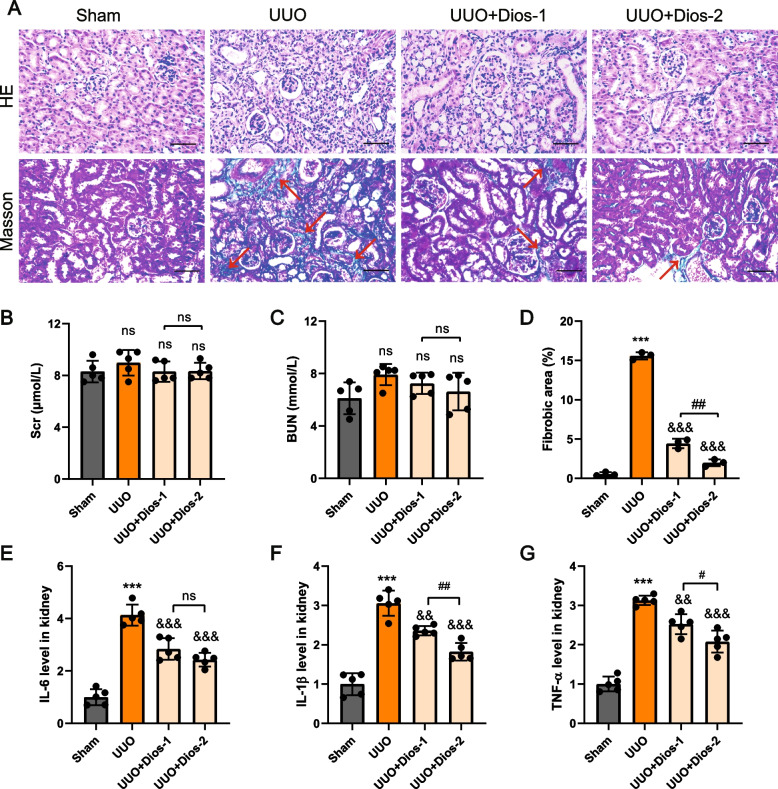
Diosmin attenuates UUO-induced renal injury and inflammation. **A** Representative images of HE staining and Masson staining of kidney tissue. **B** Scr and (**C**) BUN values for the different groups. **D** Quantification of Masson staining-positive areas. **E** Detection of protein expression levels of IL-6, (**F**) IL-1β and (**G**) TNF-α in the serum using Elisa. ^*^p < 0.05, and ^***^p < 0.001 *versus* the Sham group; ^&&^*p* < 0.01, and ^&&&^*p* < 0.001 *versus* the UUO group; ^#^*p* < 0.05, ^##^*p* < 0.01; ns = no significance; Scr = serum creatinine; BUN = blood urea nitrogen; Scale bar = 100 μm. Dios-1 = Diosmin 50 mg/kg; Dios-2 = Diosmin 100 mg

To assess the potential impact of Diosmin on renal injury, we examined tissue staining, renal function, and the expression of inflammatory cytokines in four groups of mice. H&E staining showed diffuse infiltration of inflammatory cells, degeneration and necrosis of renal tubular epithelial cells, and renal tubular dilatation in the kidney tissue of UUO mice (Fig. 1A). Diosmin treatment improved renal histologic damages significantly. Inflammatory cytokine levels were subsequently measured in the kidneys and blood of mice. Enzyme-linked immunosorbent assay showed that Diosmin inhibited the levels of IL-1β, IL-6 and TNF-α in the kidneys of mice in the UUO group (Fig. 1F-H). Furthermore, staining with Masson trichrome suggested that Diosmin could attenuate UUO-induced hyperplasia of interstitial collagen fibers significantly in the fibrotic kidneys of mice (Fig. 1A, D). There was no significant difference in Scr, BUN (Fig. 1B, C), and the kidney weight: bodyweight ratio (Fig. S1) among the four groups. In addition, Diosmin had no significant effect on serum levels of ALT, AST, or ALP (Fig. S2).

### Diosmin inhibits the UUO-induced collagen deposition and fibrosis

**Fig. 2:**
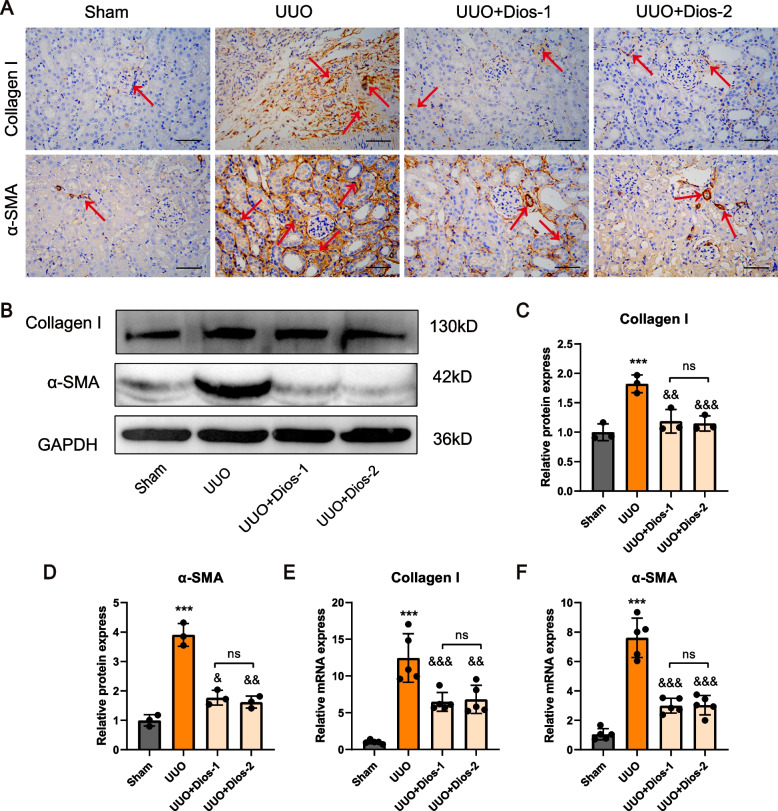
Diosmin inhibits the UUO-induced collagen deposition and fibrosis. **A** Representative images of collagen I and α-SMA IHC staining. **B** Representative western blot analysis of collagen I and α-SMA. **C** Quantitative analysis of collagen I and **D** α-SMA. **E** The mRNA expression levels of collagen I and **F** α-SMA in the left kidney of mice. ^***^p < 0.0001 *versus* the Sham group; ^&&^*p* < 0.01, and ^&&&^*p* < 0.001 *versus* the UUO group; ^#^*p* < 0.05, ^##^*p* < 0.01 and ^###^*p* < 0.001; ns = no significance; Scale bar = 100 μm. Dios-1 = Diosmin 50 mg/kg; Dios-2 = Diosmin 100 mg/kg

Sustained activation of inflammatory cytokines caused by UUO leads to the conversion of fibroblasts in the renal interstitium into myofibroblasts. The latter secrete collagen fibers that are non-degradable, which leads to the accumulation and deposition of large amounts of extracellular matrix collagen, thereby destroying the renal tissue structure. To assess the potential impact of Diosmin on renal fibrosis, we examined renal fibrosis markers in four groups of mice. Immunohistochemical staining showed a significant increase in protein expression of collagen I and a-SMA in the kidneys of mice in the UUO group (Fig. 2A). Whereas Diosmin treatment inhibited the expression of fibrosis-marking proteins. In addition, Western blotting also proved consistent results (Fig. 2B-D). RT-qPCR showed that the levels of collagen I and a-SMA mRNA were suppressed after D intervention (Fig. 2E, F). These results indicated that Diosmin alleviated UUO-induced kidney injury, collagen production, and renal fibrosis in mice.

### Diosmin inhibits TGF-β1 induced inflammation and fibrosis in HK-2 cells

**Fig. 3:**
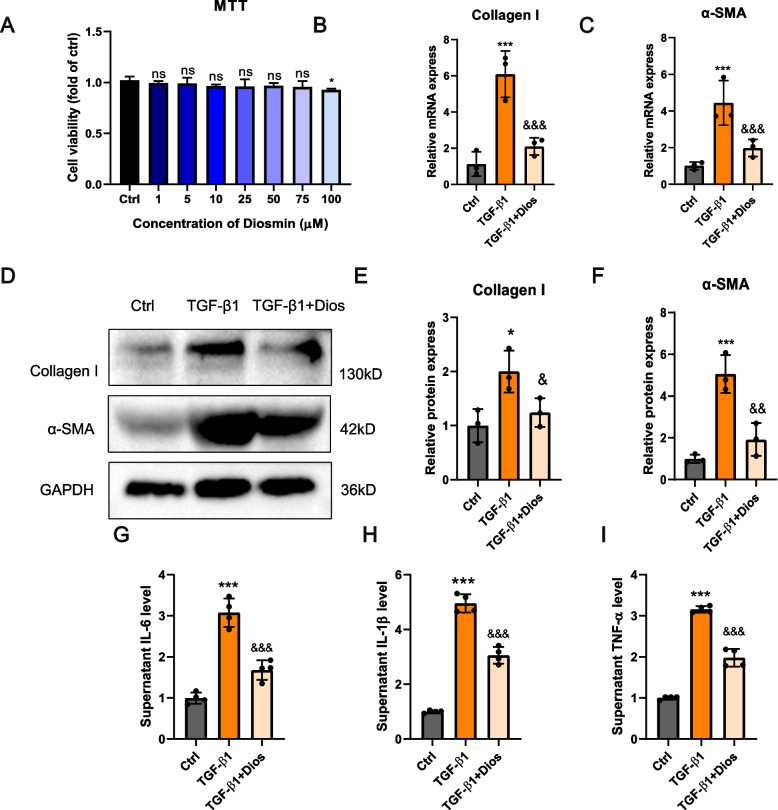
Diosmin reduces TGF-β1 induced inflammation and fibrosis in HK-2 cells. **A** MTT assay detects the toxicity of Diosmin on HK-2 cells. **B** The mRNA expression levels of collagen I and (**C**) α-SMA. **D** Representative western blot analysis of collagen I and α-SMA. **E** Quantitative analysis of collagen I and (**F**) α-SMA. **G** The levels of IL-6, (**H**) IL-1β and (**I**) TNF-α in the supernatant. ^*^p < 0.05, and ^***^p < 0.001 *versus* the control (DMSO) group; ^&^*p* < 0.05, ^&&^*p* < 0.01, and ^&&&^*p* < 0.001 *versus* the TGF-β1 group; ns = no significance; Ctrl = control; Dios = 75uM

We further investigated the anti-fibrotic effect of Diosmin on human renal tubular epithelial (HK-2) cells. Diosmin treatment (0, 1, 5, 10, 25, 50, and 75 μM) of HK-2 cells did not produce a significant inhibitory effect on their viability (Fig. 3A). Cell viability was inhibited slightly at a Diosmin concentration of 100 μM. Therefore, we chose to treat cells with Diosmin at a concentration of 75 μM. HK-2 cells treated with TGF-β1 (5 ng/mL) increased the mRNA and protein expression of collagen I and α-SMA. Diosmin inhibited the transcription and translation the protein expression of α-SMA and collagen I in HK-2 cells stimulated by TGF-β1 (Fig. 3B-F). Consistent with the in vivo results, Diosmin inhibited secretion of IL-1β, IL-6, and TNF-α in HK-2 cell supernatants following TGF-β1 stimulation (Fig. 3G-I). Taken together, Diosmin attenuated fibrosis and inflammation in HK-2 cells under TGF-β1 stimulation.

### Diosmin increases SIRT3 expression in vivo and in vitro

**Fig. 4:**
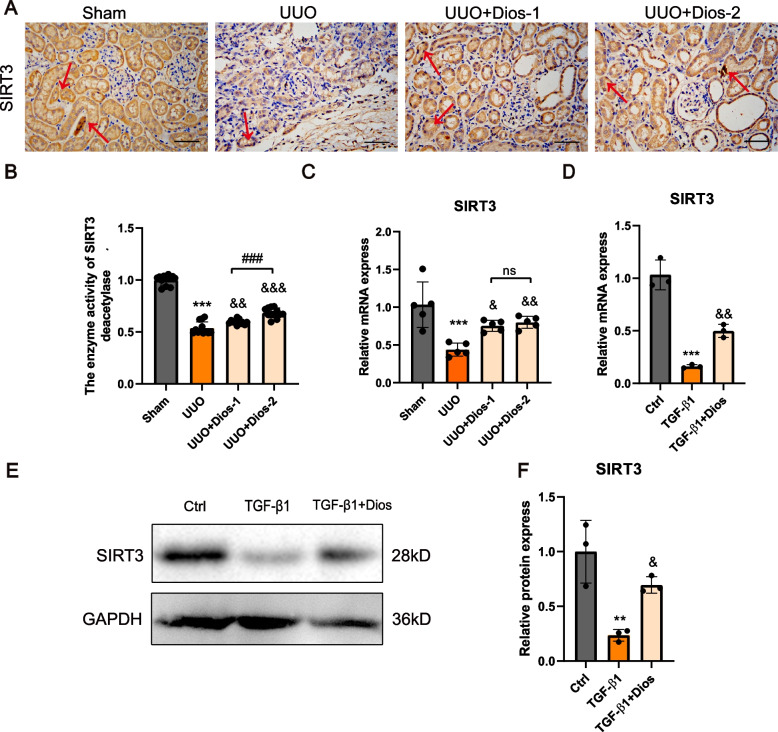
Diosmin increases SIRT3 expression in vivo and in vitro. **A** Representative images of SIRT3 IHC staining. **B** The enzyme activity of SIRT3 in left kidney of different groups of mice. **C** The mRNA expression levels of SIRT3 in the left kidney and (**D**) HK-2. (**E**) Representative western blot analysis and (**F**) quantitative analysis in the HK-2. ^**^p < 0.01, and ***p < 0.001 *versus* the Sham or the control (DMSO) group; ^&^*p* < 0.05, ^&&^*p* < 0.01, and ^&&&^*p* < 0.001 *versus* UUO or TGF-β1 group; ^###^*p* < 0.001; ns = no significance; Ctrl = control; Dios = 75uM; Dios-1 = Diosmin 50 mg/kg; Dios-2 = Diosmin 100 mg/kg

We found that protein expression of SIRT3 was significantly reduced in the kidneys of the UUO group, while Diosmin was able to partially restore SIRT3 expression (Fig. 4A). Western blotting also indicated consistent results (Fig. 4E, F). Therefore, we further explored the effect of Diosmin on SIRT3 activity. UUO treatment decreased SIRT3 activity in kidney, whereas Diosmin increased SIRT3 activity significantly in the fibrotic kidneys (Fig. 4B). Furthermore, the effect of Diosmin on SIRT3 expression was examined in vivo and in vitro. Treatment with UUO and TGF-β1 decreased the protein and mRNA expression of SIRT3 in kidney tissue and HK-2 cells, but intervention with Diosmin reversed this phenomenon (Fig. 4C, D). Thus, Diosmin increased SIRT3 expression and activity.

### Deletion of SIRT3 eliminates the anti-inflammatory effects of Diosmin

**Fig. 5:**
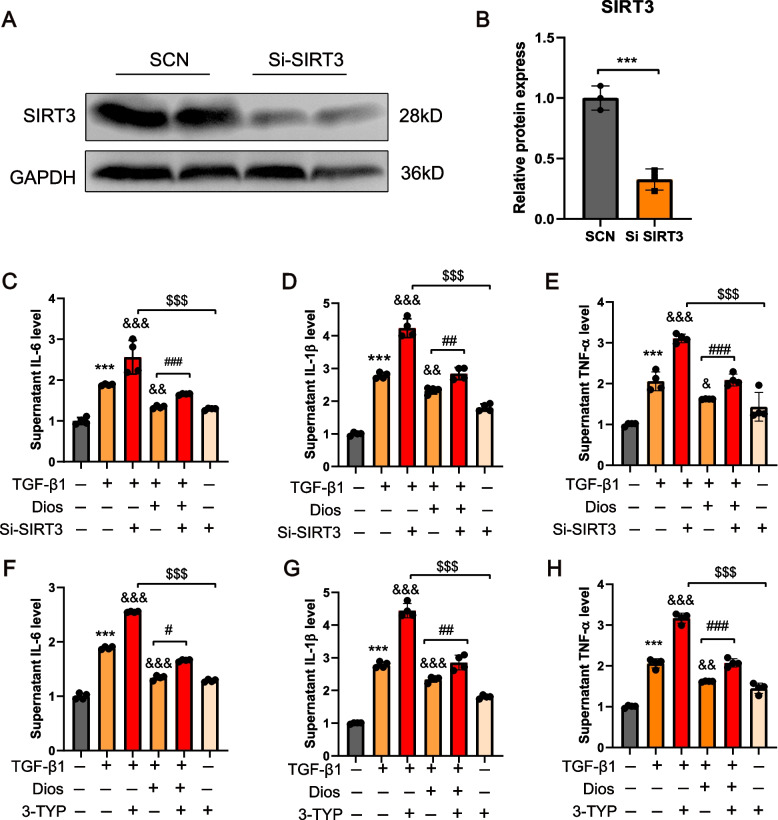
Deletion of SIRT3 eliminates the anti-inflammatory effects of Diosmin. **A**, **B** Western blotting for the transfection efficiency. **C**-**E** The effect of SIRT3 SiRNA on the secretion of IL-1β, IL-6, and TNF-α regulated by Diosmin in the supernatant of the culture medium of HK-2 cells. **F-H** The effect of 3-TYP (SIRT3 selective inhibitor) on the secretion of IL-1β, IL-6, and TNF-α regulated by Diosmin in the supernatant of the culture medium of HK-2 cells. ^**^p < 0.01, and ^***^p < 0.001 *versus* the control (DMSO) group; ^&^*p* < 0.05, ^&&^*p* < 0.01, and ^&&&^*p* < 0.001 *versus* the TGF-β1 alone group; ^#^*p* < 0.05, ^##^*p* < 0.01 and ^###^*p* < 0.001 *versus* the Dios + TGF-β1 group; ^$$$^*p* < 0.001 *versus* the SIRT3 siRNA alone group; ns = no significanc

We treated HK-2 cells with SIRT3 siRNA or 3YTP (SIRT3-selective inhibitor) and investigated the regulation of SIRT3 in the anti-inflammatory effect of Diosmin. Western blotting was used to detect transfection efficiency (Fig. 5A, B). SIRT3 siRNA significantly attenuated the inhibitory effect of Diosmin on inflammation in TGF-β1-induced HK-2 cells (Fig. 5C-E). Furthermore, identical results were obtained using 3-TYP (Fig. 5F-H). However, 3-TYP alone had no detectable effect on levels of inflammatory cytokines compared with controls. In addition, the 3-TYP + TGF-β1 group and SIRT3 siRNA + TGF-β1 significantly increased the levels of inflammatory cytokines compared with those using 3-TYP alone or SIRT3 siRNA alone, thereby indicating that 3-TYP or SIRT3 siRNA was effective only in pathologic conditions.

### Diosmin ameliorates renal fibrosis by regulating SIRT3-mediated NF-κB p65 nuclear translocation

**Fig. 6:**
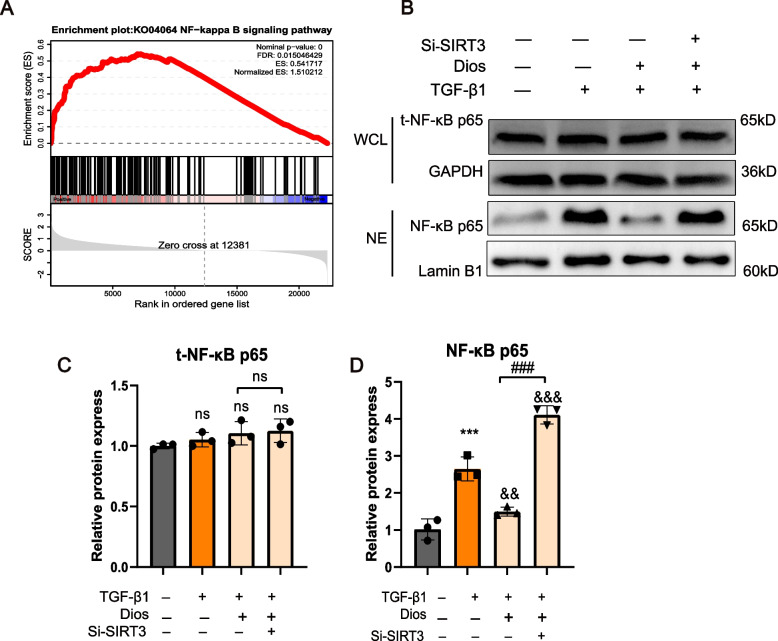
Diosmin ameliorates renal fibrosis by regulating SIRT3-mediated NF-κB p65 nuclear translocation. **A** The enrichment plot of NF-κB signaling pathway. **B** Representative western blot analysis of the expression of total NF-κB P65 and nuclear NF-κB p65 in HK-2 cells. **C** Quantitative analysis of total NF-κB p65 and (**D**) nuclear NF-κB p65. ^***^p < 0.001 *versus* the control (DMSO) group; ^&&&^*p* < 0.001 *versus* the TGF-β1 alone group; ^###^*p* < 0.001 *versus* the Dios + TGF-β1 group; ns = no significance; WCL = whole cell; NE = nucleus; t-NF-κB p65 = total NF-κB p65

**Fig. 7:**
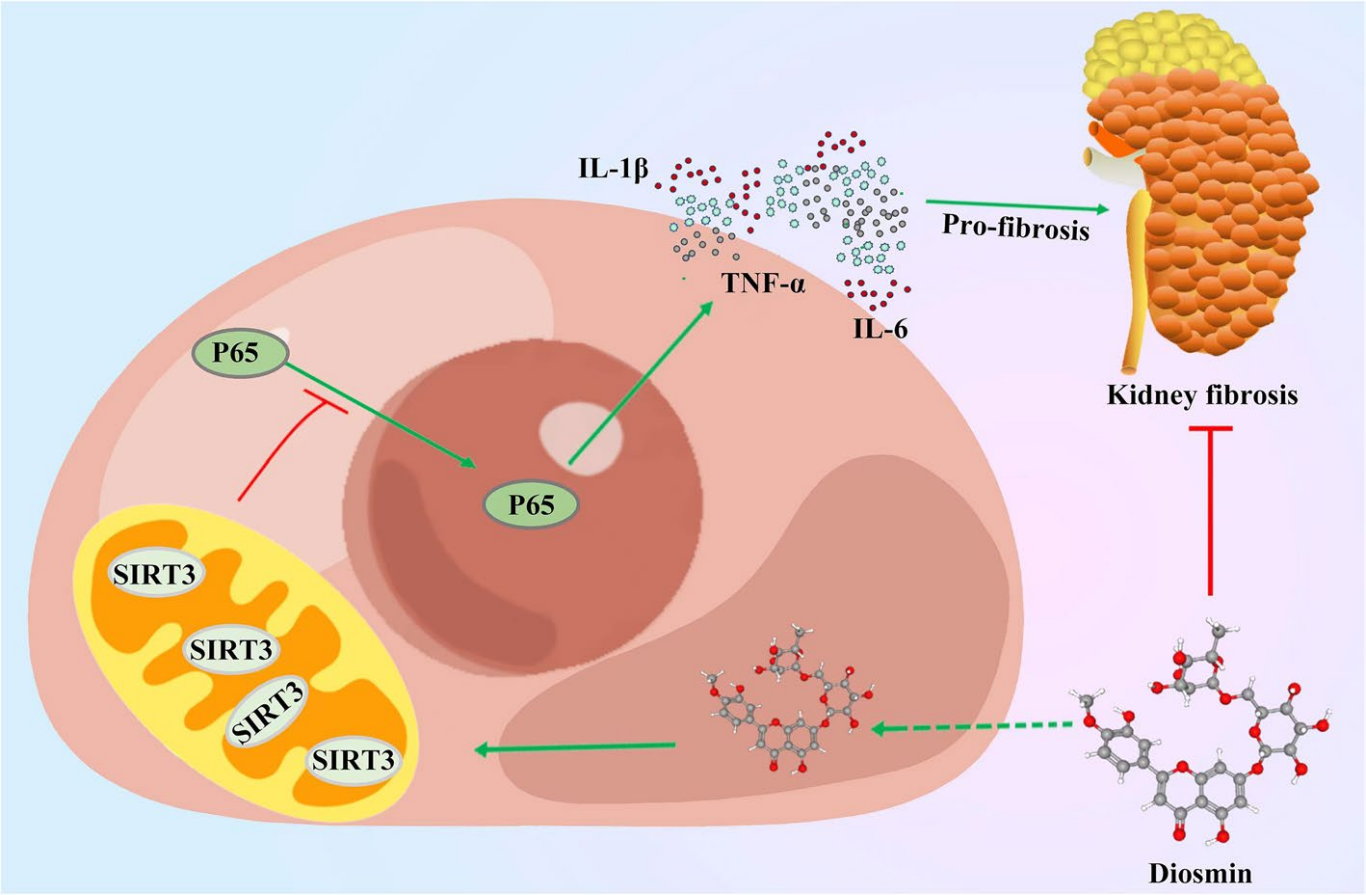
Schema of the underlying mechanism anti-inflammatory effect of Diosmin in renal fibrosis. Diosmin performs promotes the expression and activity of SIRT3, then inhibits nuclear translocation of NF-κB p65 to reduce inflammatory cytokines (TNF-α, IL-6, and IL-1β) levels. Therefore, Diosmin exerts the anti-fibrotic effect. Altogether, Diosmin attenuates renal fibrosis mainly by suppressing NF-κB p65-inflammation pathway, which is dependent on induction of SIRT3 expression

To further explore the mechanism of Diosmin inhibition of fibrosis, we analyzed the differentially expressed genes in the kidneys of mice from UUO and Diosmin intervention groups using RNA sequencing (RNA-seq). We found that the NF-κB signaling pathway was significantly down-regulated after Diosmin intervention compared to UUO mice (Fig. 6A). Hence, we investigated how SIRT3 regulates inflammation by focusing on the NF-κB signaling pathway in stimulated HK-2 cells. TGF-β1 significantly increased the nuclear expression of NF-κB p65 in HK-2 cells. However, TGF-β1 and Diosmin had no significant effect on the total expression of NF-κB p65 in HK-2 cells (Fig. 6B-D). Diosmin inhibited the nuclear expression of NF-κB p65 in HK-2 cells. After knockdown of SIRT3 expression, the inhibitory effect of Diosmin on NF-κB p65 was attenuated significantly. Taken together, SIRT3 mediates Diosmin-induced anti-fibrotic effects by reducing the nuclear expression of NF-κB p65 in HK-2 cells after treatment (Fig. 7).

## Discussion

Anti-inflammatory therapy is an effective strategy for treating fibrosis [29, 30]. We demonstrated that Diosmin prevented UUO- or TGF-β1-induced renal fibrosis mainly by inhibiting inflammation. Decreased levels of inflammatory cytokines were documented. In particular, SIRT3 was involved in the anti-inflammatory effect of Diosmin-induced renal fibrosis. Diosmin promoted SIRT3 expression, and deletion of SIRT3 abolished the inhibitory effect of Diosmin upon inflammation. Interestingly, SIRT3 was involved in the regulation of nuclear expression of NF-κB p65, and silencing SIRT3 expression increased the nuclear expression of NF-κB p65 in HK-2 cells. Thus, our data clearly demonstrate that anti-inflammatory effects against Diosmin-induced renal fibrosis can be promoted by regulating SIRT3-mediated nuclear NF-κB p65 expression.

Renal fibrosis is considered to be an inflammation-related disease [31, 32]. Inflammatory cells secrete many inflammatory cytokines and further infiltrate renal tissue, making the kidney in an “inflammatory microenvironment”. Without efficacious treatment, persistent inflammatory stimulation can further aggravate irreversible kidney damage. NF-κB p65 is a key regulator mediating the regulation of inflammation. Phosphorylation and nuclear translocation of p65 are the main modes of activation of the NF-kB pathway. Therefore, p65-nuclear localization sequence is often used to reflect the expression of active p65. Several studies have shown that regulation of nuclear NF-κB p65 expression can control inflammation effectively [33–35]. Studies have reported that Liuweiwuling tablets can alleviate bile-duct ligation-induced liver fibrosis mainly by inhibiting the expression of the inflammatory cytokines IL-1β1, TNF-α, and IL-6 [36]. The anti-hepatic-fibrosis effect of the novel oridonin analog CYD0618 is accomplished mainly by blockade of the nuclear translocation of NF-κB p65 and preventing the phosphorylation of the NF-κB inhibitory protein IκBα [37]. Recently, Chen et al. [37] found that, in a carbon tetrachloride-induced model of liver fibrosis in rats, increased expression of SIRT3 attenuated inflammation and fibrosis by inhibiting NF-κB p65 nuclear translocation. Consistent with the results of other studies, our results indicated that TGF-β1 increased the expression of nuclear NF-κB p65, whereas Diosmin decreased the expression of nuclear NF-κB p65 significantly. Therefore, the anti-inflammatory effect of Diosmin is also dependent upon NF-κB.

SIRT3 is a promising target for treatment of inflammation-related diseases [38, 39]. Kurundkar et al. [40] reported that SIRT3 expression was reduced significantly in mouse lungs upon activation of the NLR family pyrin domain during lipopolysaccharide-induced acute lung injury. Activation of SIRT3 with vinferin reduced activation of the NLRP3 inflammasome and production of inflammatory cytokines, and attenuated lung-tissue damage. However, the protection mentioned above was abolished in SIRT3-knockout mice [40]. Zhang et al. [41] demonstrated that melatonin could exhibit anti-inflammatory, anti-oxidative stress, and anti-apoptotic renoprotective effects via activation of SIRT3 in models of acute renal failure in vivo and in vitro. Wu et al. [42] showed that Rhein has antioxidant capacity and anti-fibrosis effects in a model of chronic kidney disease because it can activate the SIRT3/Fxo3α signaling pathway. Palomer et al. [43] found that SIRT3-knockout mice developed myocardial inflammation and fibrosis. In contrast, SIRT3 overexpression in the cardiomyocytes of neonatal rats prevented (at least in part) TNF-α-induced inflammatory responses and attenuated fibrotic responses. We found that UUO or TGF-β1 decreased the activity and expression of SIRT3 significantly, while increasing the secretion of inflammatory cytokines and the expression of NF-κB p65 in activated hematopoietic stem cells. In addition, SIRT3 overexpression reduced the expression of NF-κB p65, suggesting that SIRT3 mediates the regulation of TGF-β1-induced inflammation mainly by regulating the expression of NF-κB p65 during renal fibrosis, data which are consistent with the results of other reports [44, 45]. Therefore, our data suggest that SIRT3 may be a potential therapeutic target of inflammation-related diseases such as renal fibrosis. In the absence of TGF-β1 stimulation, 3-TYP alone or SIRT3 siRNA alone did not have a significant effect on the secretion of inflammatory cytokines, but they significantly promoted secretion of inflammatory cytokines. This phenomenon may be due to TGF-β1 stimulation leading to the formation of some pathologic microenvironments characterized by excessive secretion of inflammatory cytokines, whereas SIRT3 siRNA or 3-TYP exerted their effects only in such pathologic microenvironments. SIRT3 siRNA or 3-TYP enhanced TGF-β1-induced secretion of inflammatory cytokines.

As a flavonoid compound, Diosmin has anti-inflammatory, anti-oxidative stress, anti-fibrotic, and other effects [21–23]. Tahir et al. [46] found that Diosmin could protect against ethanol-induced liver injury by attenuating the inflammatory response and regulating activation of TNF-α and NF-κB. Yang et al. created a mouse model of renal ischemia by non-traumatic microvascular clipping of the left renal pedicle combined with resection of the right renal pedicle. After perfusion, Diosmin treatment inhibited nuclear NF-κB and mitochondrial apoptotic pathways and activated the Nrf2/HO-1 pathway to protect the kidneys [47]. Fattori et al. [48] applied Diosmin to block NF-κB activation in the treatment of lipopolysaccharide-induced inflammatory pain and peritonitis in mice. We found that Diosmin can suppress the level of inflammation and inhibit the progression of renal fibrosis by upregulating the expression of SIRT3.

Diosmin may increase SIRT3 expression and activity through multiple pathways. However, exactly how Diosmin regulates SIRT3 is not known. Our research team will further explore the potential mechanisms. Furthermore, to investigate further the role of SIRT3 in Diosmin-induced anti-fibrotic effects, we will use SIRT3-knockout mice in future studies. The Smad protein [49, 50] has been studied widely as a transcription factor and key intracellular effector of TGF-β1. Smad4 and Smad3 are pro-fibrotic, whereas Smad2 and Smad7 protect against fibrosis [51, 52]. As a key mediator in the pathogenesis of renal fibrosis, TGF-β1 can activate Smad-dependent and non-dependent pathways to express its biological activity. Therefore, we will explore the function of TGF-β1/Smad signaling in Diosmin-induced renal fibrosis in future studies.

However, it is important to acknowledge certain limitations in our study. First, our research primarily focused on preclinical models, and the transition to clinical studies in human subjects is warranted to confirm the safety and efficacy of Diosmin in a real-world setting. Second, while our investigation provides insights into the mechanism of action of Diosmin, additional in-depth studies are needed to elucidate the complete signaling pathways involved. Furthermore, a more comprehensive assessment of the potential adverse effects and side effects of Diosmin should be undertaken as part of future research, considering its translational potential.

## Conclusions

Diosmin was found to attenuate renal fibrosis mainly by inhibiting inflammation. The anti-inflammatory effect of Diosmin was dependent on SIRT3-mediated nuclear expression of NF-κB p65. Our results provide new insights into the molecular mechanisms by which Diosmin regulates inflammation-related diseases and could be a natural candidate for the treatment of renal fibrosis.

### Supplementary Information

**Additional file 1:** **Figure S1.** The kidney weight / bodyweight between the four groups of mice. **Figure S2.** Serum ALT, AST, and ALP levels in four groups of mice.

**Additional file 2.**

## Data Availability

The data in this study are available from the corresponding author request.
