# Supplementary material for: Diosmin ameliorates renal fibrosis through inhibition of inflammation by regulating SIRT3-mediated NF-κB p65 nuclear translocation
Source: BMC Complement Med Ther. 2024 Jan 9;24:29. doi: 10.1186/s12906-023-04330-z (PMC10777592; doi:10.1186/s12906-023-04330-z)
Supplement: Supplementary file 1 — Additional file 1: Figure S1. The kidney weight / bodyweight between the four groups of mice. Figure S2. Serum ALT, AST, and ALP levels in four groups of mice. [file 12906_2023_4330_MOESM1_ESM.docx]

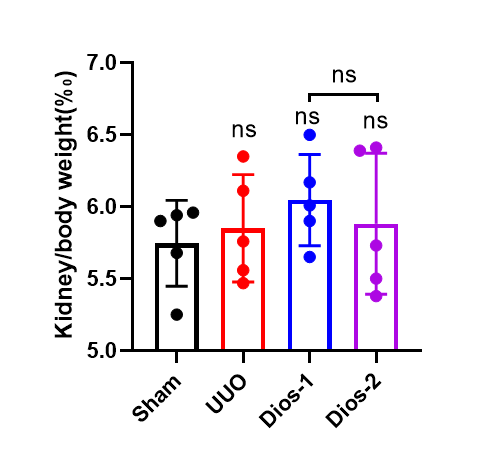


**Figure S1** The kidney weight / bodyweight between the four groups of mice. ns, no significance; Dios-1, UUO+Diosmin 50 mg/kg; Dios-2, UUO+Diosmin 100 mg/kg.


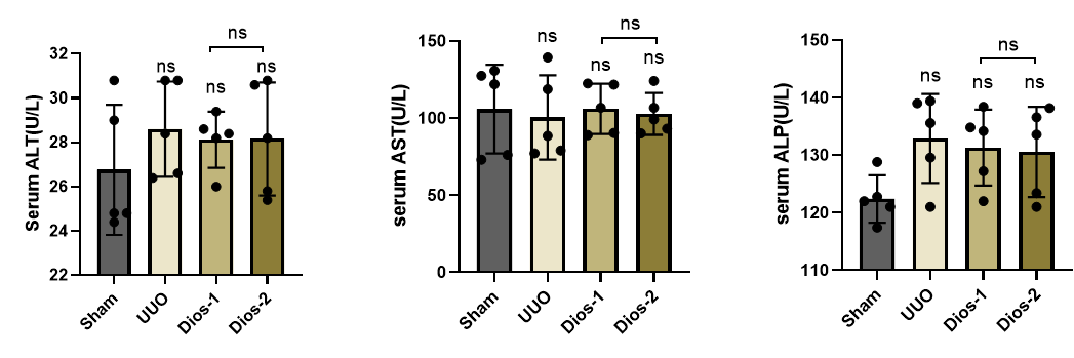


**Figure S2** Serum ALT, AST, and ALP levels in four groups of mice. ns, no significance; Dios-1, UUO+Diosmin 50 mg/kg; Dios-2, UUO+Diosmin 100 mg/kg.
