# Supplementary material for: Diosmin ameliorates renal fibrosis through inhibition of inflammation by regulating SIRT3-mediated NF-κB p65 nuclear translocation
Source: BMC Complement Med Ther. 2024 Jan 9;24:29. doi: 10.1186/s12906-023-04330-z (PMC10777592; doi:10.1186/s12906-023-04330-z)

Fig.2B

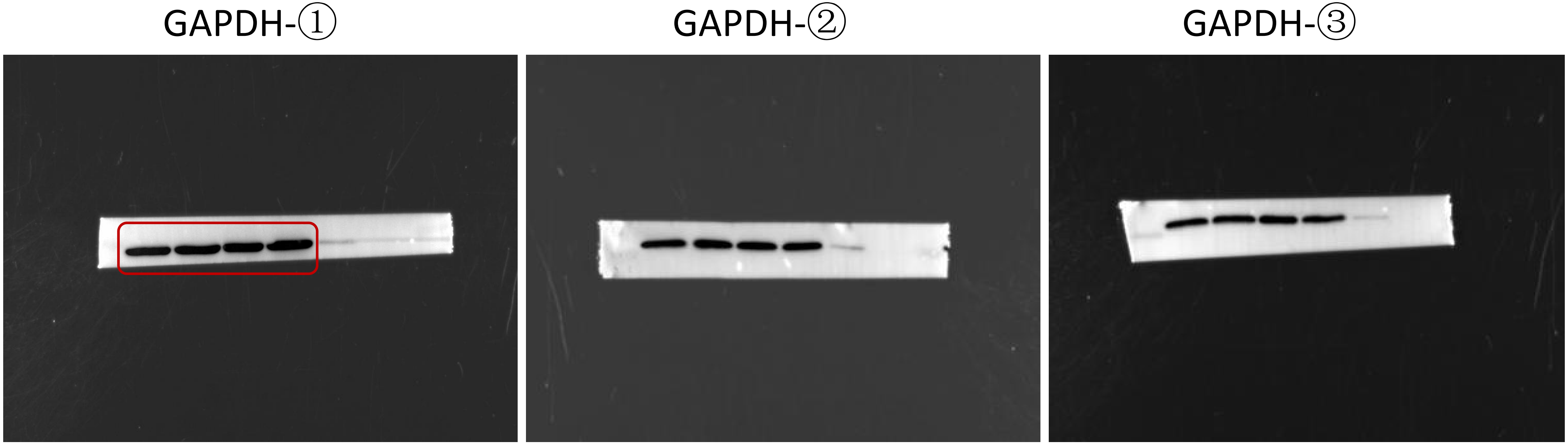

*Part of the the blots were cut prior to hybridisation with antibodies during blotting.

Fig.2B

Collagen1-①

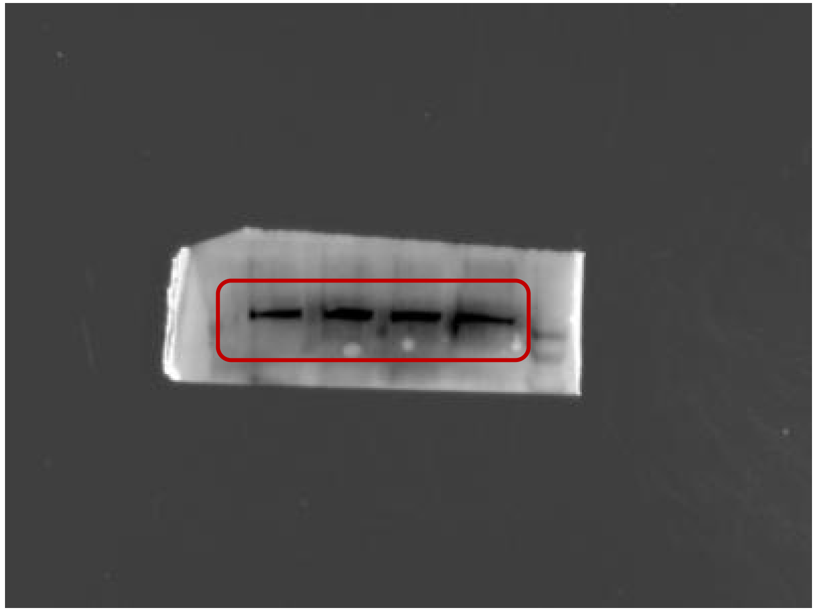

Collagen1-②

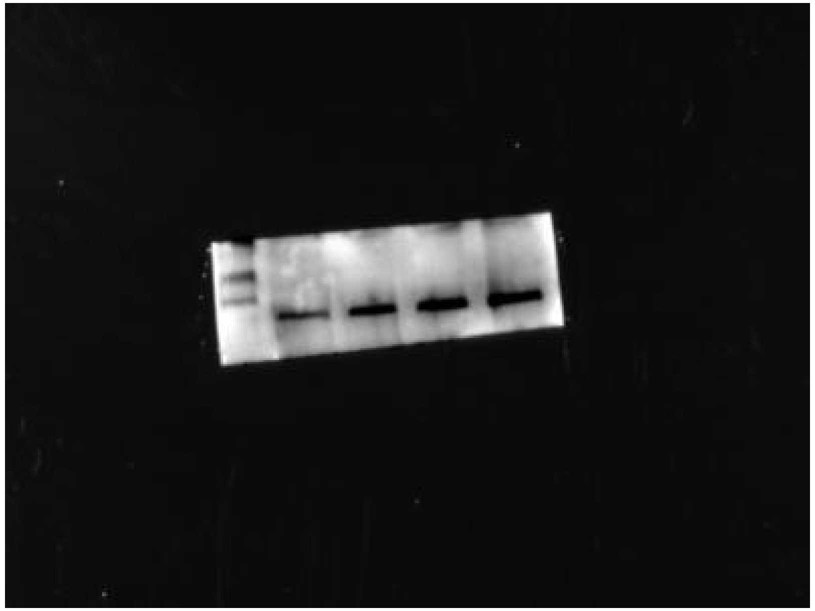

Collagen1-③

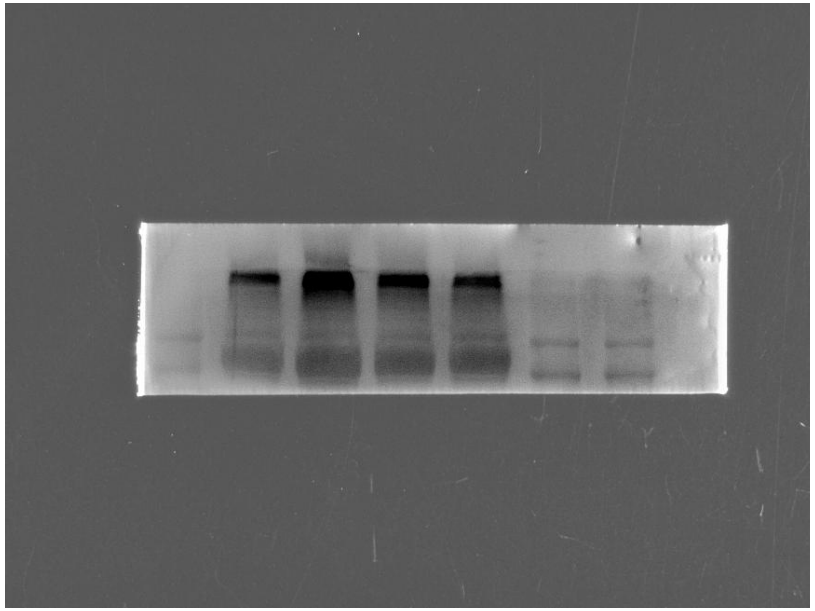

Fig.2B

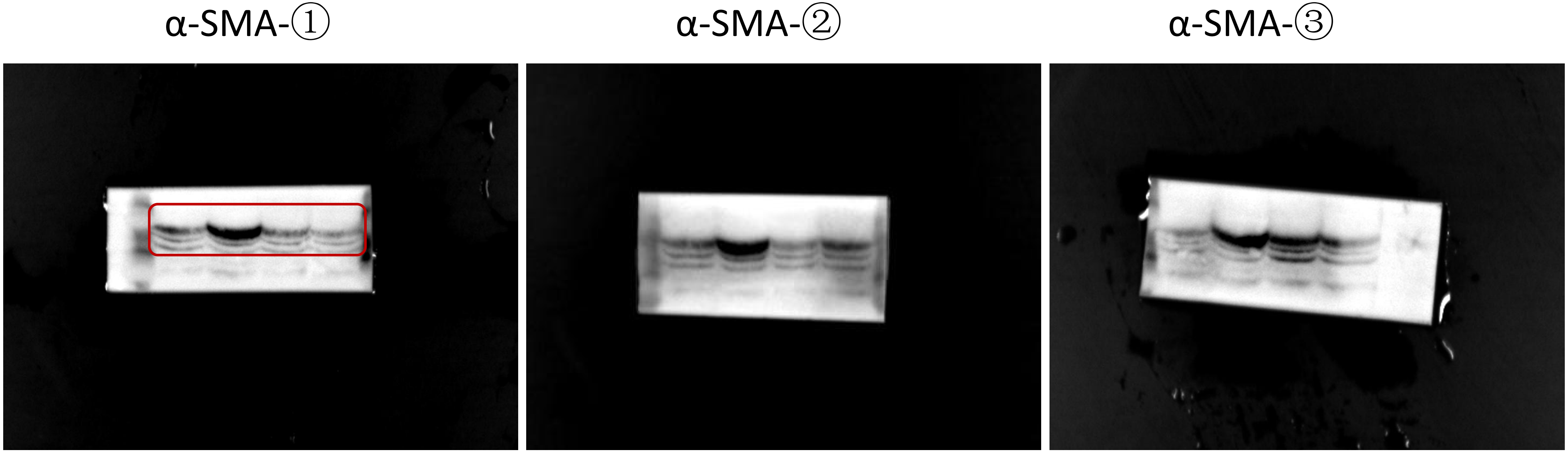

Fig.3D

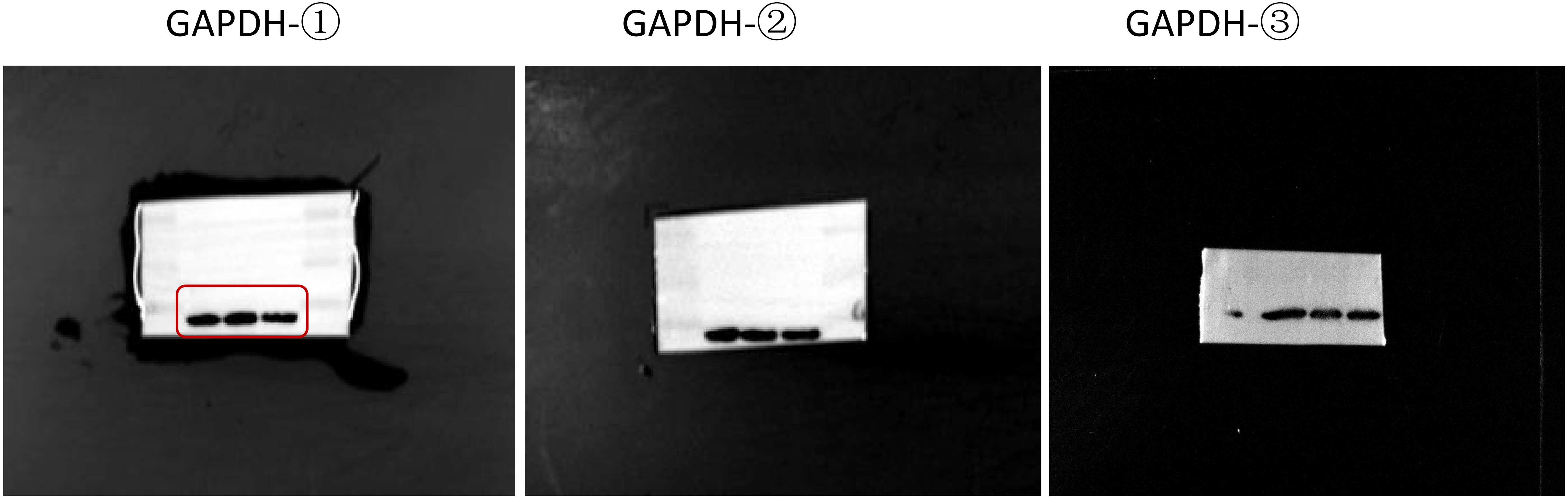

Fig.3D

Collagen1-① Collagen1-② Collagen1-③

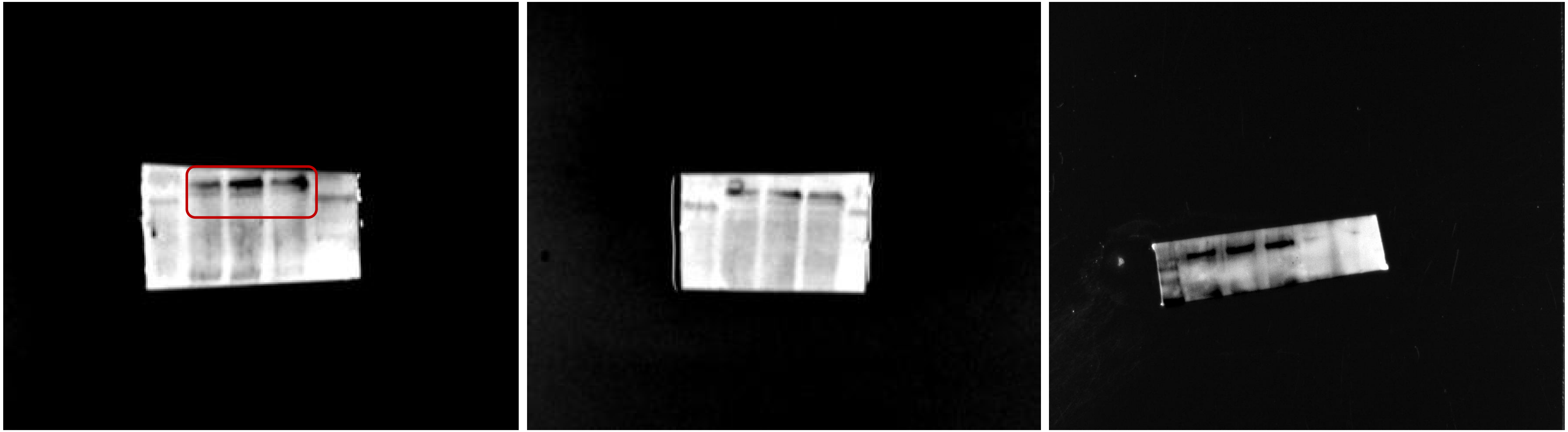

Fig.3D

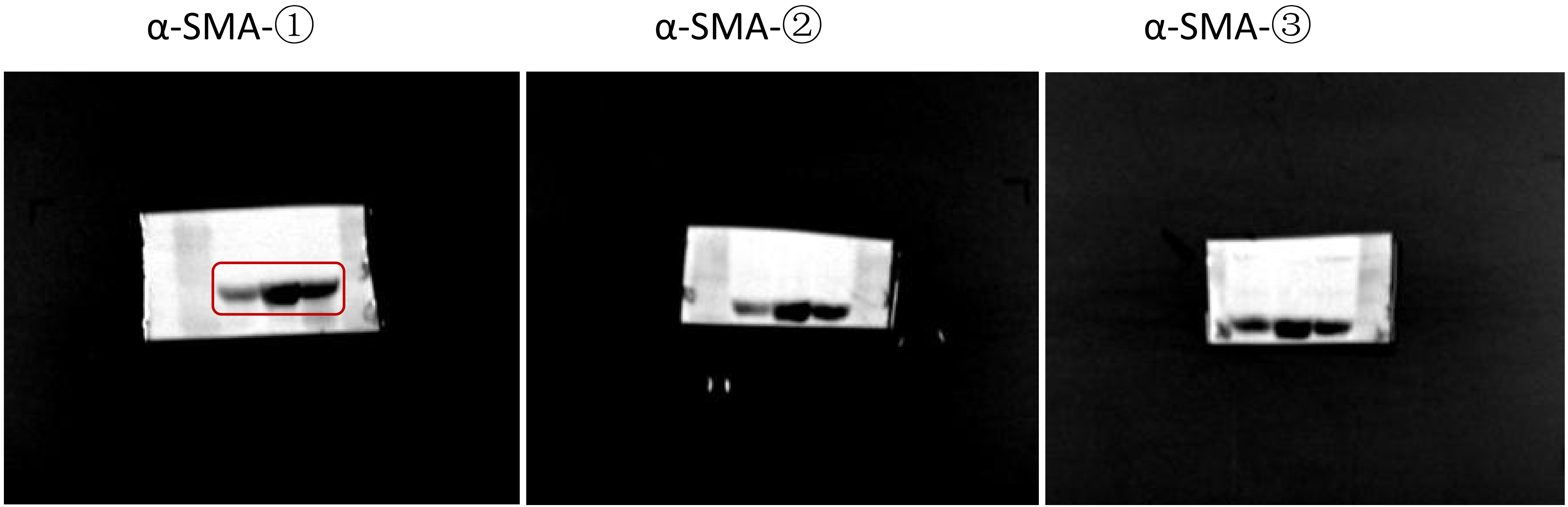

Fig.4E

GAPDH-①

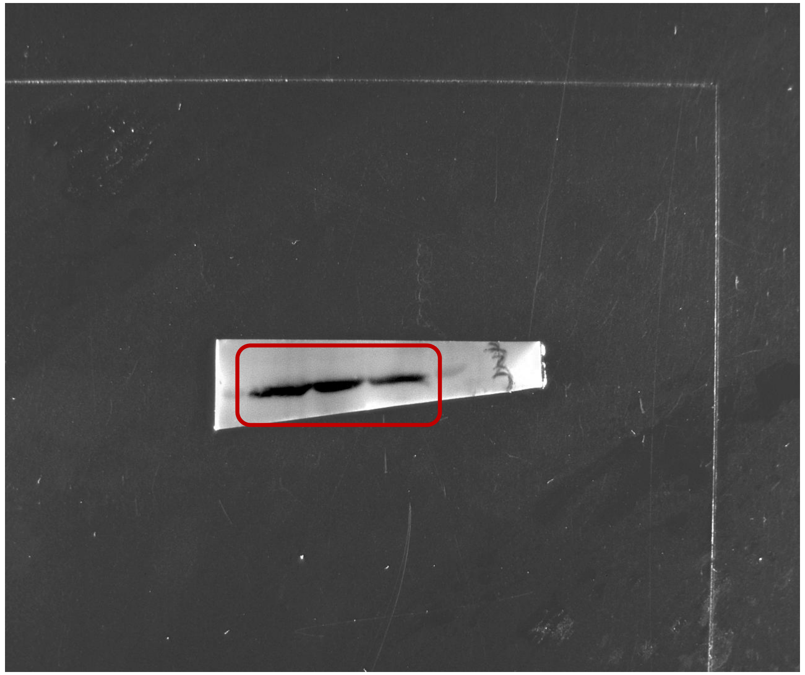

GAPDH-②

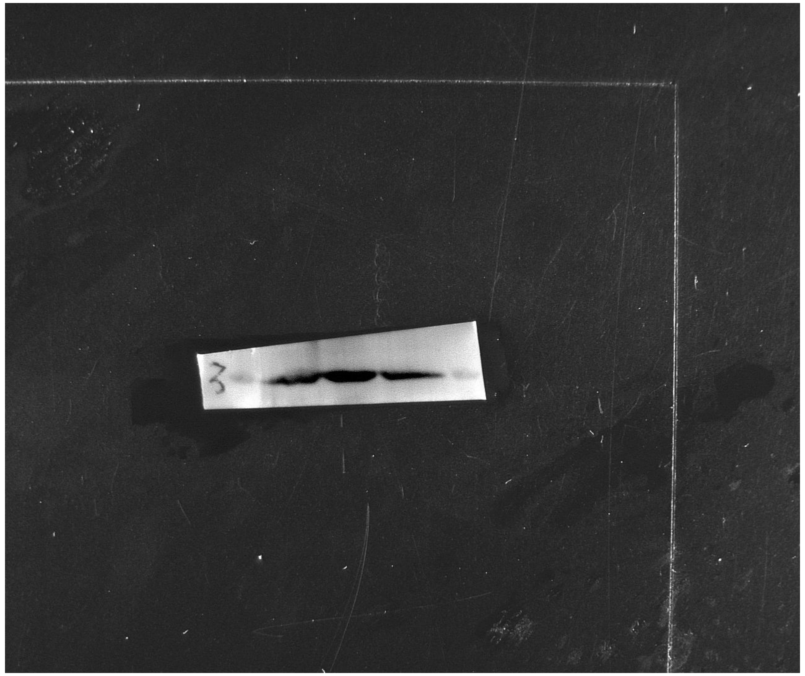

GAPDH-③

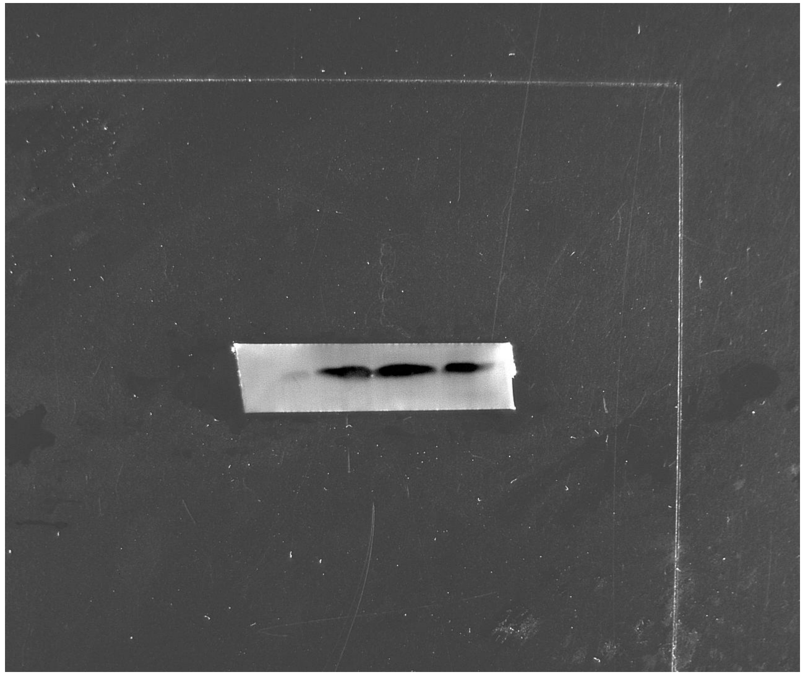

Fig.4E

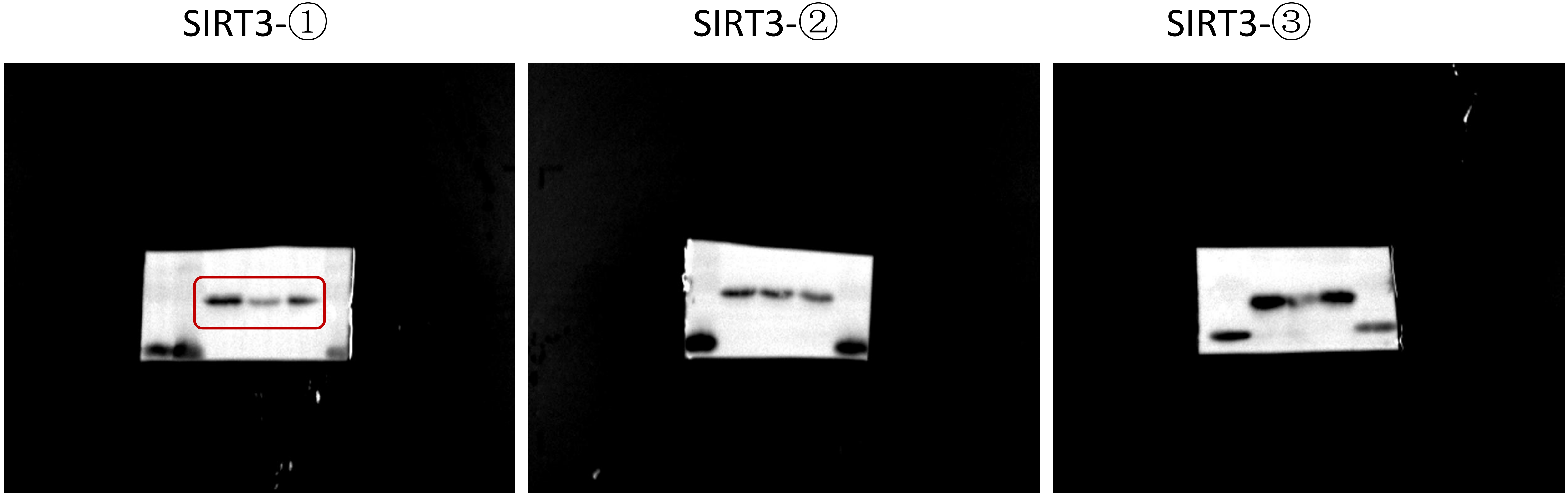

Fig.5A

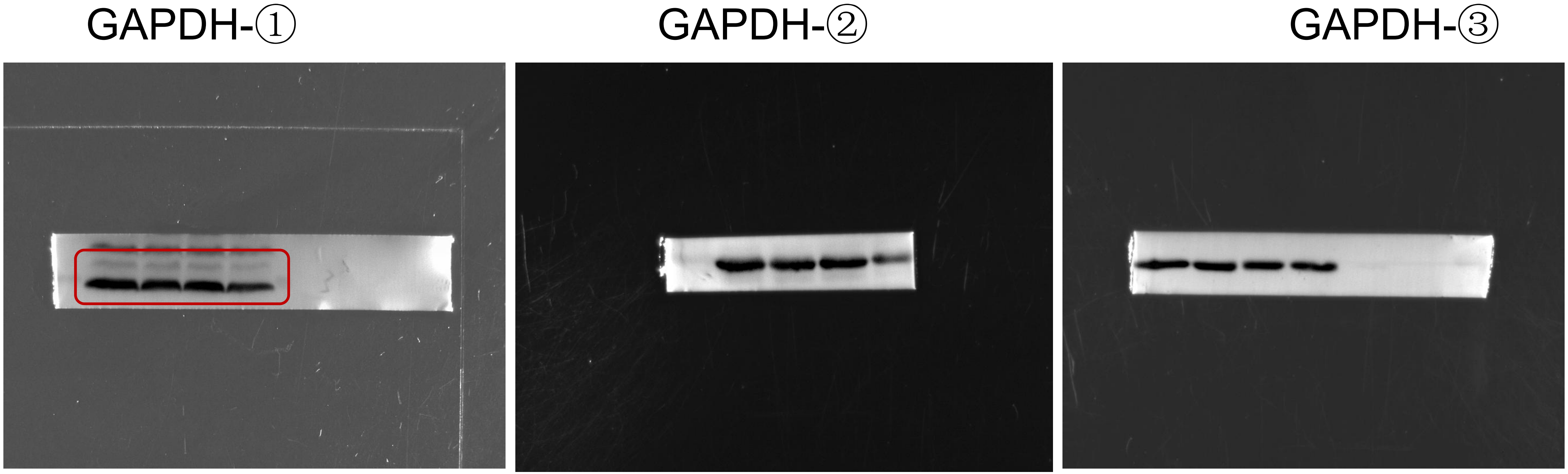

Fig.5A

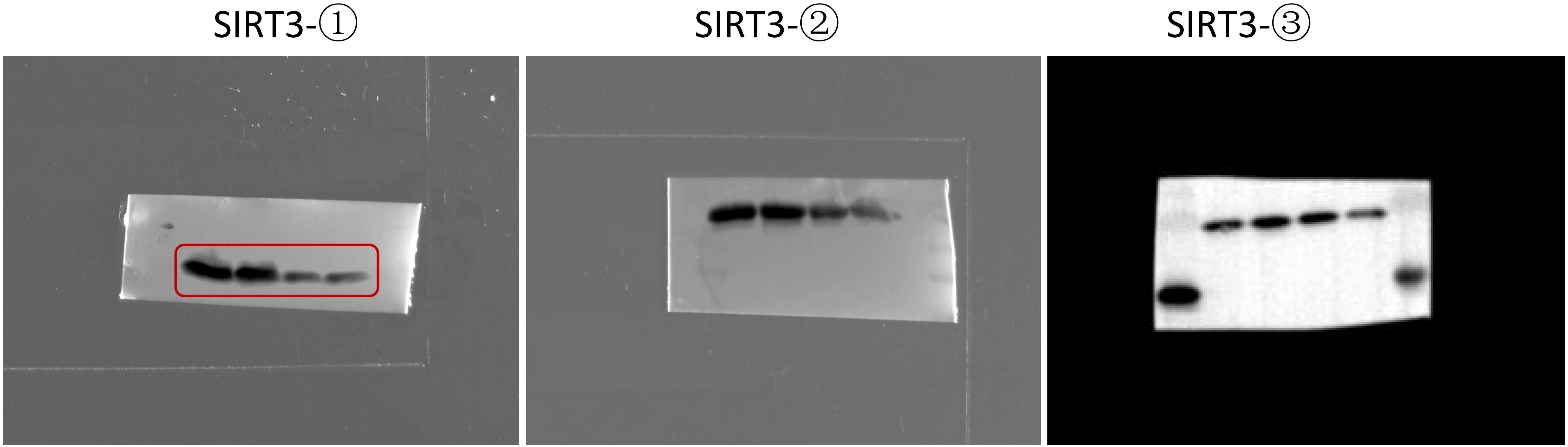

Fig.6B

t-NF-κB p65-①

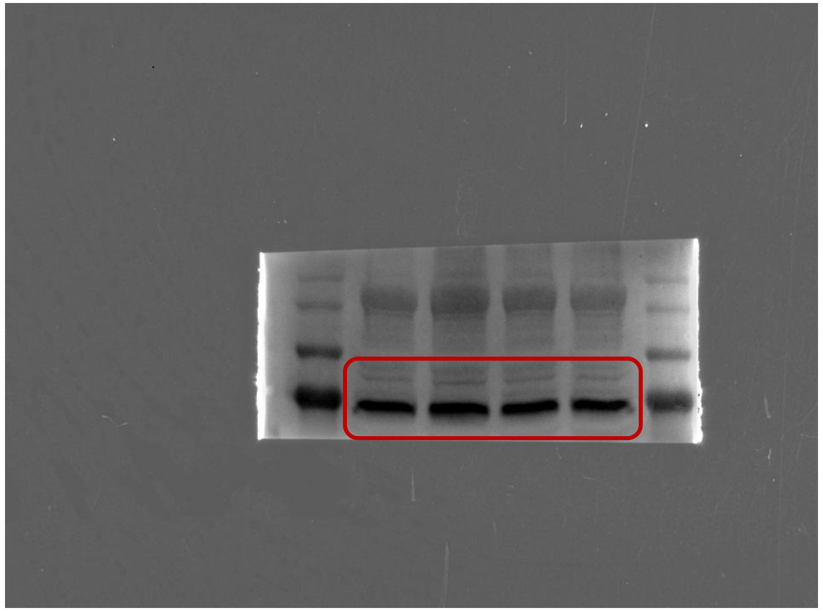

t-NF-κB p65-②

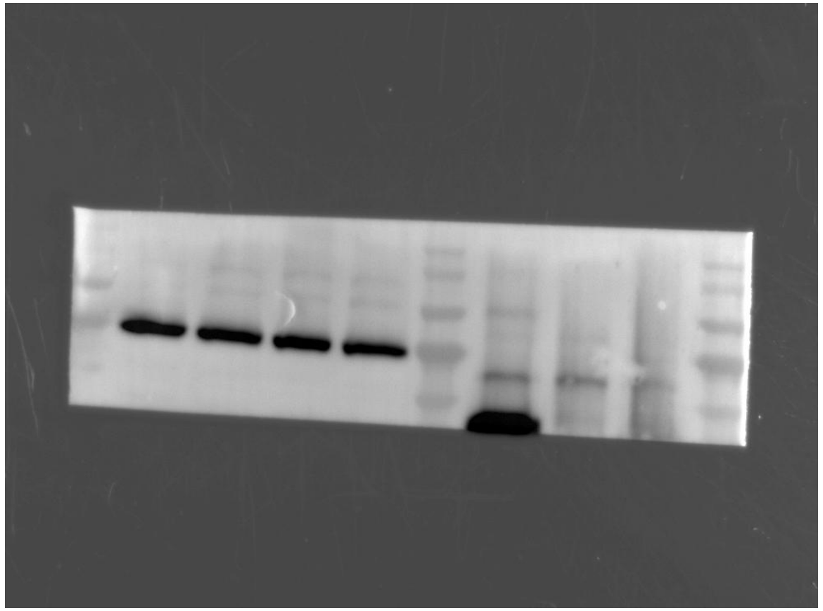

t-NF-κB p65-③

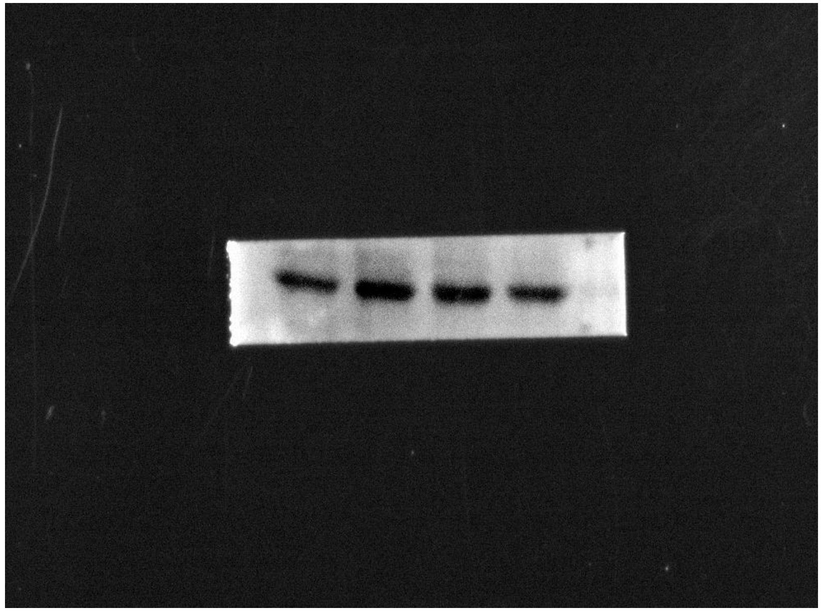

Fig.6B

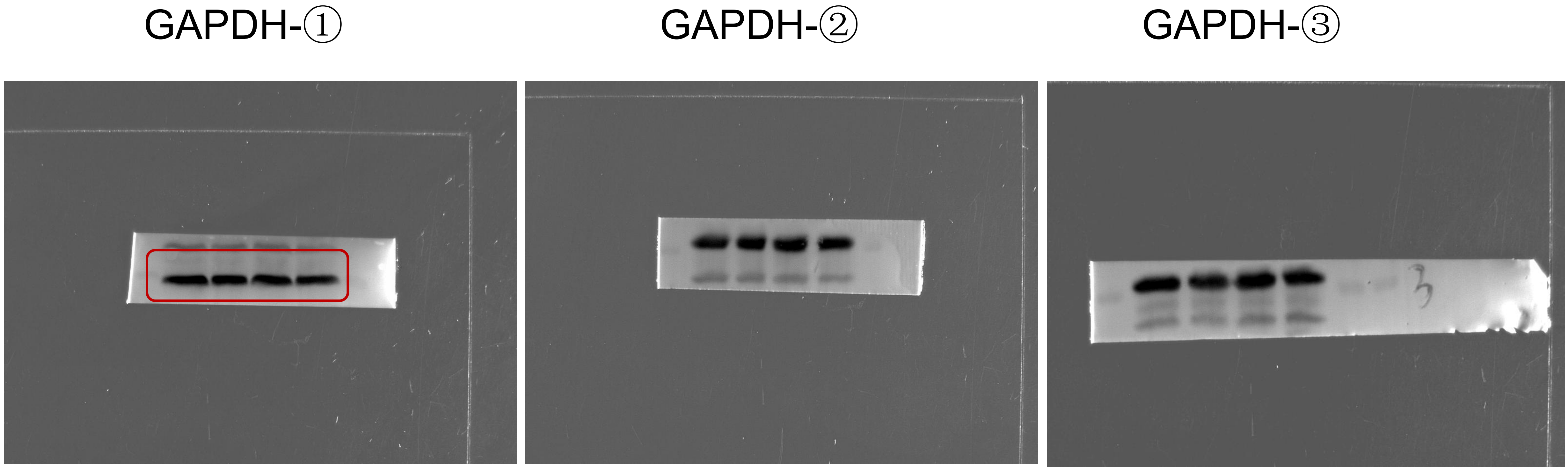

Fig.6B

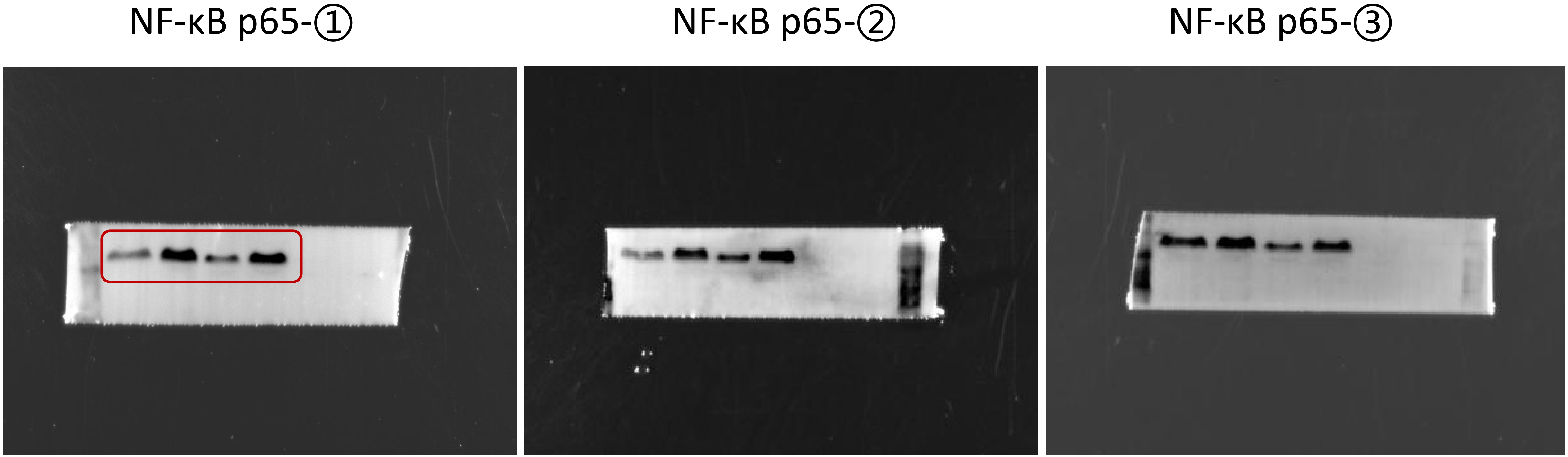

Fig.6B

Lamin B1-① Lamin B1-② Lamin B1-③

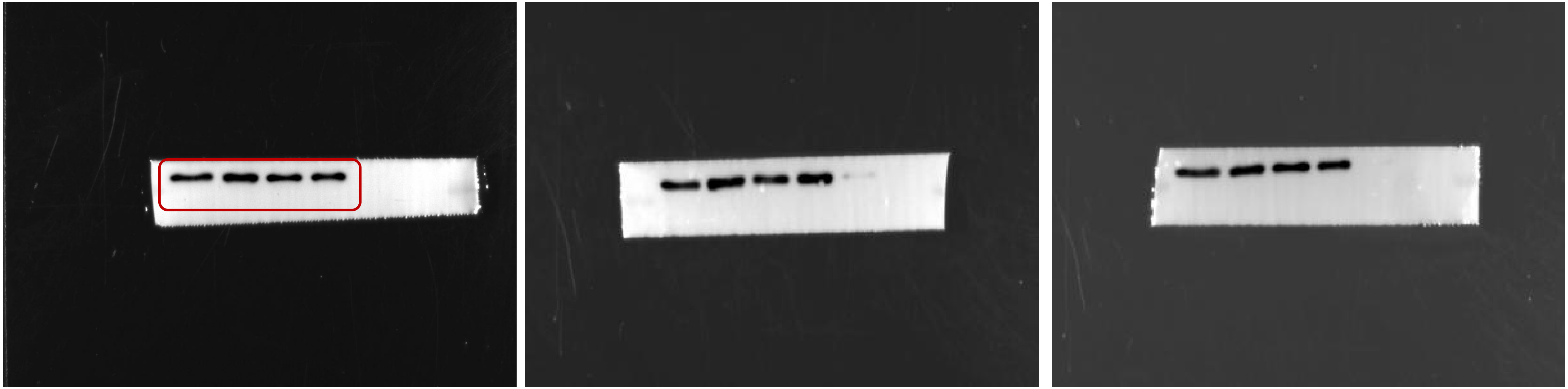

Supplement: Supplementary file 2 — Additional file 2. [file 12906_2023_4330_MOESM2_ESM.pdf]
